# Multi-Tiered Observation and Response Charts: Prevalence and Incidence of Triggers, Modifications and Calls, to Acutely Deteriorating Adult Patients

**DOI:** 10.1371/journal.pone.0145339

**Published:** 2015-12-30

**Authors:** Arthas Flabouris, Savvy Nandal, Luke Vater, Katerina Flabouris, Alice O’Connell, Campbell Thompson

**Affiliations:** 1 Intensive Care Unit, Royal Adelaide Hospital and Discipline of Acute Care Medicine, University of Adelaide, Adelaide, SA, Australia; 2 Department of Medicine, Royal Adelaide Hospital, Adelaide, SA, Australia; 3 School of Medicine, Faculty of Health Sciences, University of Adelaide, Adelaide, SA, Australia; 4 Intensive Care Unit, Royal Adelaide Hospital, and School of Medicine, University of Adelaide, Adelaide, SA, Australia; 5 Department of Medicine, Royal Adelaide Hospital and School of Medicine, University of Adelaide, Adelaide, SA, Australia; King Abdullah International Medical Research Center, SAUDI ARABIA

## Abstract

**Background:**

Observation charts are the primary tool for recording patient vital signs. They have a critical role in documenting triggers for a multi-tiered escalation response to the deteriorating patient. The objectives of this study were to ascertain the prevalence and incidence of triggers, trigger modifications and escalation response (Call) amongst general medical and surgical inpatients following the introduction of an observation and response chart (ORC).

**Methods:**

Prospective (prevalence), over two 24-hour periods, and retrospective (incidence), over entire hospital stay, observational study of documented patient observations intended to trigger one of three escalation responses, being a MER—Medical Emergency Response [highest tier], MDT—Multidisciplinary Team [admitting team], or Nurse—senior ward nurse [lowest tier] response amongst adult general medical and surgical patients.

**Results:**

Prevalence: 416 patients, 321 (77.2%) being medical admissions, median age 76 years (IQR 62, 85) and 95 (22.8%) Not for Resuscitation (NFR). Overall, 193 (46.4%) patients had a Trigger, being 17 (4.1%) MER, 45 (10.8%) MDT and 178 (42.8%) Nurse triggers. 60 (14.4%) patients had a Call, and 72 (17.3%) a modified Trigger. Incidence: 206 patients, of similar age, of whom 166 (80.5%) had a Trigger, 122 (59.2%) a Call, and 91 (44.2%) a modified Trigger. Prevalence and incidence of failure to Call was 33.2% and 68% of patients, respectively, particular for Nurse Triggers (26.7% and 62.1%, respectively). The number of Modifications, Calls, and failure to Call, correlated with the number of Triggers (0.912 [p<0.01], 0.631 [p<0.01], 0.988 [p<0.01]).

**Conclusion:**

Within a multi-tiered response system for the detection and response to the deteriorating patient Triggers, their Modifications and failure to Call are common, particularly within the lower tiers of escalation. The number of Triggers and their Modifications may erode the structure, compliance, and potential efficacy of structured observation and response charts within a multi-tiered response system.

## Introduction

Patient clinical deterioration, whether detected by abnormal vital signs or staff concerns, occurs commonly prior to cardiac arrest, unanticipated intensive care unit (ICU) admission or death [1,2]. Failure to respond to clinical deterioration is common [3], and the type of response to the deteriorating patient can adversely affect patient outcome [4].

These circumstances provide the basis for Rapid Response Systems, designed to detect and respond to clinical deterioration [5]. The Australian Commission on Safety and Quality in Health Care (ACSQHC) has designated the recognition, and response to, clinical deterioration as a National Safety and Quality Health Service Standards (Standard 9) for adoption throughout Australia [6].

Afferent Limb Failure (ALF) is failure to respond to clinical deterioration despite documented predefined triggers for activation of a Rapid Response Team (RRT). ALF occurs in approximately 22% of RRT calls [3], and is associated with increased risk of patient mortality or unanticipated ICU admission [3,7]. Contributors to ALF are varied and complex, and include inaccuracy and/or poor documentation of patient vital signs [8,9], errors in judgment [10], and not calling for help [11]. Minimizing ALF may increase RRT activation, which itself is associated with improved patient outcomes [12].

Patient observation charts are the primary tool for recording vital signs. A factor that can contribute to poor recording, and interpretation, of vital signs, is observation chart design [13,14]. Thus there is a strong emphasis on the standardised design, and use of, observation charts that not only identify patients who are deteriorating (“track”), but also guide the type of clinical escalation in response to predefined criteria (“triggers”). Observation and response charts, based upon human factor design, are now recommended in tracking and identifying patients at risk of an adverse event [6]. Observation and response charts also include the option to record modifications to vital sign thresholds. This is consistent with the ACSQHC Consensus Statement [6]. Despite their widespread adoption, there is very little evidence for the clinical utility of observation and response charts, or the relative contribution of their key components, especially within a multi-tiered system.

By examining a track-and-trigger patient observation chart that provides the basis for a multi-tiered escalation response to the deteriorating patient, we sought to ascertain the incidence and prevalence of documented clinical triggers, modifications to these triggers, and the expected escalation response amongst a cohort of hospital inpatients.

## Materials and Methods

### Study design and Patients

Observational study with a prospective prevalence component, conducted over two separate 24-hours periods, and a retrospective incidence component, of a convenience (non-randomly selected) sample of recently discharged patients, encompassing the patient’s entire hospital stay.

The setting was the Royal Adelaide Hospital, a metropolitan, tertiary referral, university-affiliated, adult acute hospital of approximately 620 beds in Adelaide, South Australia. Patients are admitted under a range of services, including general medical, specialty medical, general surgical and specialty surgical teams. Patients admitted under general medicine are typically those requiring management of complex, chronic and multisystem disorders who present with an acute medical problem or decompensated chronic disorder [15]. Patients admitted under general surgery are those with acute surgical illness, typically involving abdominal conditions [16].

All study patients were inpatients admitted to a general medical or general surgical team. General medical or general surgical patients who were within the, ICU, Step down/High Dependency Unit (SDU) or in the operating theatre/recovery, during the two prevalence observation periods (Saturday, 14^th^ September 2013 and Sunday 27^th^ October 2013) were excluded.

A standardised patient observation and response chart, based upon ACSQHC guidelines, has been in use since July 2013 [17]. It delineates three different coloured “zones”, which are expected to prompt any one of three tiers of escalated response to review a deteriorating patient, based upon which zone the vital sign falls into. The chart contains instructions for staff as to which of the three tiers of response to call. Charts also have sections for documenting modifications so as either not to trigger, or to alter, the expected escalation response.

We recorded patient demographics, admitting unit, resuscitation status, and from the observation charts, documented vital signs intended to trigger (a Trigger) any one of three escalation response (a Call) ranging from a patient review from a senior ward nurse (Nurse [lowest level]), to the admitting medical and nursing staff as a multidisciplinary team (MDT) to a medical emergency response team (MER [highest level], being two acute medical unit doctors, two ICU nurses and when requested an ICU doctor) and whether there was documentation that the trigger need not generate an escalation as intended (a Modification). Patient de-identified data was entered onto a study-specific database (Microsoft Access) and collectively analysed using SPSS (Vs21) software.

### Outcomes and Analysis

Outcomes were the prevalence and incidence of Triggers for an escalation response, their Modifications, the type of Call and a failure to initiate a Call.

Statistical analysis involved basic descriptive statistics, including medians (25^th^/75^th^ quartiles) for continuous data, Mann-Whitney for age and Chi-square analysis of categorical data. Level of significance was set at p<0.05. Analysis was based upon the Triggers collectively, as well as for the patient group. Pearson’s correlation was used to explore the correlation between Trigger numbers and that of Modifications and Calls.

The Royal Adelaide Hospital Human Research Ethics Committee approved the study (protocol number 140409). As the study involved only review of medical records, no patient consent was required.

## Results

### Prevalence

The prevalence group included 416 inpatients with a median age of 76 years (IQR 62–85). 210 patients (50.4%) were female, 321 (77.2%), general medical admissions and 95 (22.8%) had a documented Not For Resuscitation (NFR) order. Medical patients, in comparison to surgical patients were older (median age 79 [IQR 67, 86] vs 64 [48, 74] years, p<0.01), females (53.9% vs 37.9%, p = 0.02) and had an NFR order (28.3% vs 4.2%, p<0.01).

In a 24-hour period, of the 416 patients, 193 (46.4%) had at least one Trigger, 60 (14.4%) at least one Call, whilst 72 (17.3%) a Trigger that was “modified” so as not to trigger a Call (Table 1). There were multiple Triggers, Modifications and Calls for 33 (17.1%), 12 (2.9%) and 11 (2.6%) patients, respectively.

**Table 1:** Prevalence group patient demographics, admitting team, calls and modifications for each trigger tier.

	Medical Emergency Response Trigger	Multidisciplinary Team Trigger	Nurse Trigger	No Trigger	P value
Gender (Female)	8 (47.1%)	24 (53.3%	92 (51.7%)	11 (50.7%)	0.77
Age (median, IQR)	78.5 (62, 87)	77 (62.3, 86)	76 (62, 86)	75 (60, 85)	0.44
Patients with a Call	7 (1.7%)	19 (4.6%)	48 (11.5%)		<0.01
Patients with a Modification	11 (2.6%)	21 (5%)	57 (13.7%)		<0.01
Patients with a failure to Call	4 (1%)	16 (3.8%)	111 (26.7%)		<0.01
Admitting Team Medical (N = 321, 77.2% of all patients)	15 (4.7%)	28 (8.7%)	140 (43.6%)	172 (53.6%)	
Admitting Team Surgical (N = 95, 22.8% of all patients)	2 (2.1%)	17 (17.9%)	38 (40%)	51 (53.7%)	
Not For Resuscitation (N = 95, 22.8% of all patients)	5 (5.3%)	8 (8.4%)	46 (48.4%)	47 (49.5%)	

Surgical, compared to medical, patients had significantly more MDT triggers (17 [17.9%] vs 28 [8.7%], p = 0.1), a similar proportion of Nurse (38 [40%] vs 140 [43.6%], p = 0.532) and MER (2 [2.1]% vs 15 [4.7%], p = 0.27) Triggers (Table 1), and overall Calls (22 [23.2%] vs 38 [11.8%], p<0.01). The proportion of Modifications was similar between the two groups (53 [16.5%] vs 19 [20%], p = 0.43). Patients with (n = 95), compared to those without an NFR order (n = 321), had a similar proportion of Triggers (47 [49.5%] vs 145 [45.2%], p = 0.36), Calls (12 [12.6%] vs 48 [15%], p = 0.57) and Modifications (19 [20%] vs 53 [16.5%], p = 0.43).

There were a total of 559 Triggers, of which 438 (78.4%) were within a Nurse tier, 87 (15.6%) a MDT tier and 34 (6.1%) a MER tier. Overall, 220 (39.4%) Triggers were modified so as not to trigger the intended Call. Of the 438 Nurse Triggers, 156 (35.6%) were modified, whilst 41 (47.1%) MDT, and 23 (67.6%) MER Triggers were also modified.

The time of day that a Trigger was documented was similar for Nurse (median: 11:22 [IQR 8:00–16:51]), MDT (median: 12:15 [IQR 7:55–17:15]) and MER Triggers (median: 10:40 [IQR 7:15–19:55]), (p = 0.82). There was a similar proportion of Nurses, MDT and MER triggers that occurred between 1800-0800hrs (46.6%, 49.4% and 63.6%, respectively, p = 0.16)

**Fig 1:**
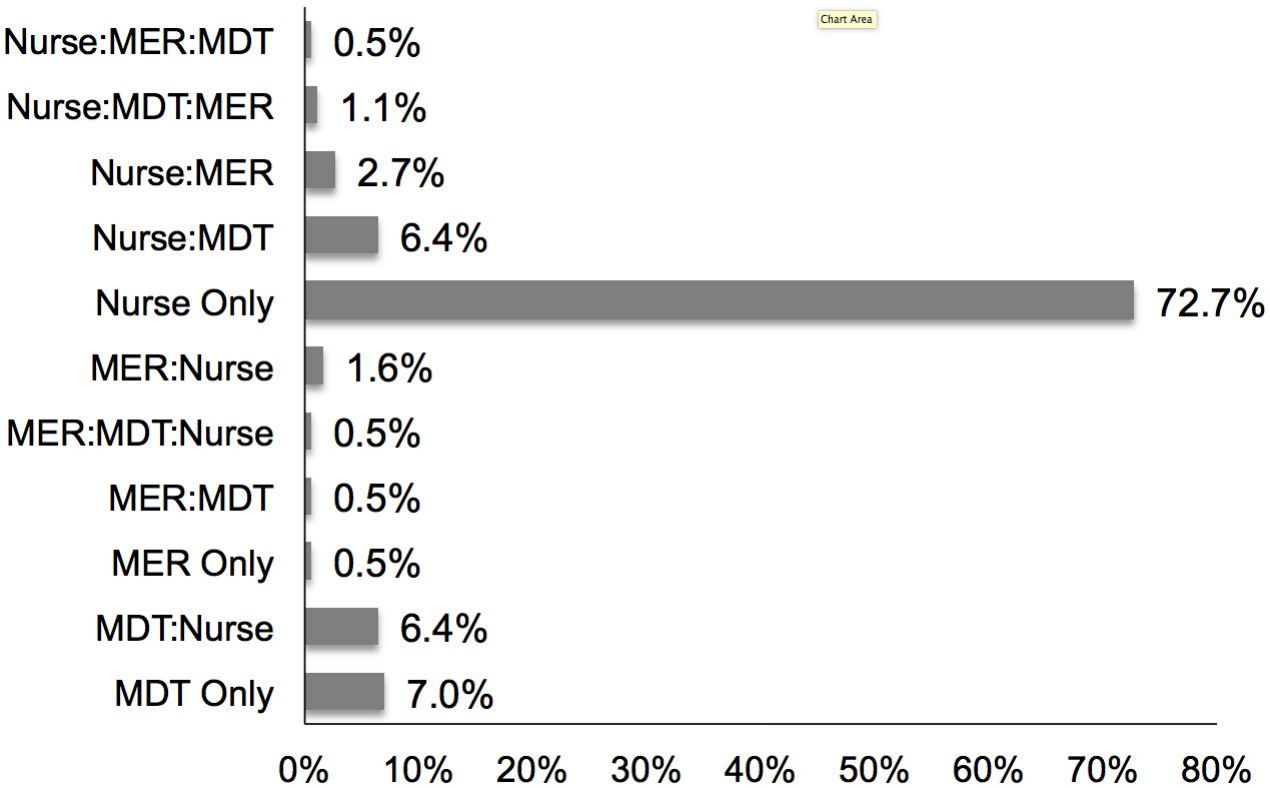
The order of occurrence of Triggers during the 24 hour prevalence periods.

The pattern of the sequence of Trigger occurrence within the 24 hour period, beginning by the first and then any subsequent Trigger, is shown in Fig 1. The majority of Triggers were Nurse only. It was not uncommon for a more extreme (ie higher tier) Trigger (for example, MER Triggers) to precede a less extreme (lower tier) Trigger.

Overall, there was a failure to initiate an escalation response (Call) as expected, for 138 (33.2%) of patients. Failure to Call was associated with a Nurse, a MDT and a MER Trigger for 111 (26.7%), 16 (3.8%) and for 4 (1%) patients, respectively. There were 282 Nurse, 46 MDT and 11 MER Triggers that were not modified, and thus expected to initiate a Call. Of these, there was a failure to initiate a Call for 213 (75.5%) Nurse Triggers, 24 (52.2%) MDT Triggers, 5 (45.5%) MER Triggers, and 242 (71.4%) of all Triggers without a modification.

### Incidence

The incidence group included 206 patients, median age of 72 years (IQR 59–85), 98 (52.4%) being female. They were of similar age (p = 0.69) and gender (p = 0.23) to the prevalence group. 135 (65.5%) were a general medical admission and 69 (33.5%) had a documented Not For Resuscitation (NFR) order at some stage during their hospital stay. Median hospital length of stay was 5 days (IQR 2–13). Amongst these patients, 29 (14.1%) died in hospital, 28 of which had an NFR order by the time of death. Medical, in comparison to surgical, patients were older (median age 80 [IQR 67, 86] vs 61 [42, 75] years, p<0.01), just as likely to be females (53.6% vs 52.1%, p = 0.95) and more likely to have an NFR order (41.5% vs 18.3%, p<0.01).

During their hospital stay, 166 (80.5%) patients had at least one Trigger, 122 (59.2%) a Call and 91 (44.2%) a Trigger that was “modified” so as not to initiate a Call (Table 2). There were multiple Triggers, Modifications and Calls for 107 (51.9%), 89 (43.2%) and 71 (34.5%) patients, respectively.

**Table 2:** Incidence group patient demographics, admitting team, calls and modifications for each trigger tier.

	Medical Emergency Response Trigger	Multidisciplinary Team Trigger	Nurse Trigger	No Trigger	P value
All patients (N = 206)	85 (41.3%)	103 (50%)	153 (74.3%)	40 (19.4%)	<0.01
Gender (Female)	53 (62.3%)	63 (61.2%)	86 (56.2%)	16 (40%)	0.09
Age (years, median, IQR)	78.5 (62, 87)	77 (62.3, 86)	76 (62, 86)	67 (51.8, 80.5)	0.06
Hospital Length of Stay (days, median, (IQR))	9 (4, 17.5)	10 (4, 19)	7 (3, 15.5)	2 (1, 4)	<0.01
Patients with a Call	62 (30.1%)	63 (30.6%)	92 (44.7%)		<0.01
Patients with a Modification	30 (14.6%)	58 (28.2%)	66 (32%)		<0.01
Patients with failure to Call	30 (14.6%)	67 (32.5%)	128 (62.1%)		<0.01
Admitting Team Medical (N = 135, 65.5% of all patients)	59 (43.7%)	69 (51.1%)	104 (77%)	25 (18.5%)	
Admitting Team Surgical (N = 71, 34.5% of all patients)	26 (36.6%)	34 (47.9%)	49 (69%)	15 (21.1%)	
Not For Resuscitation (N = 69, 33.5% of all patients)	45 (65.2%)	50 (72.5%)	57 (82.6%)	8 (11.6%)	
Hospital Discharge status Died (N = 29, 14.1% of all patients)	21 (72.4%)	23 (79.3%)	25 (86.2%)	3 (10.3%)	

**Fig 2:**
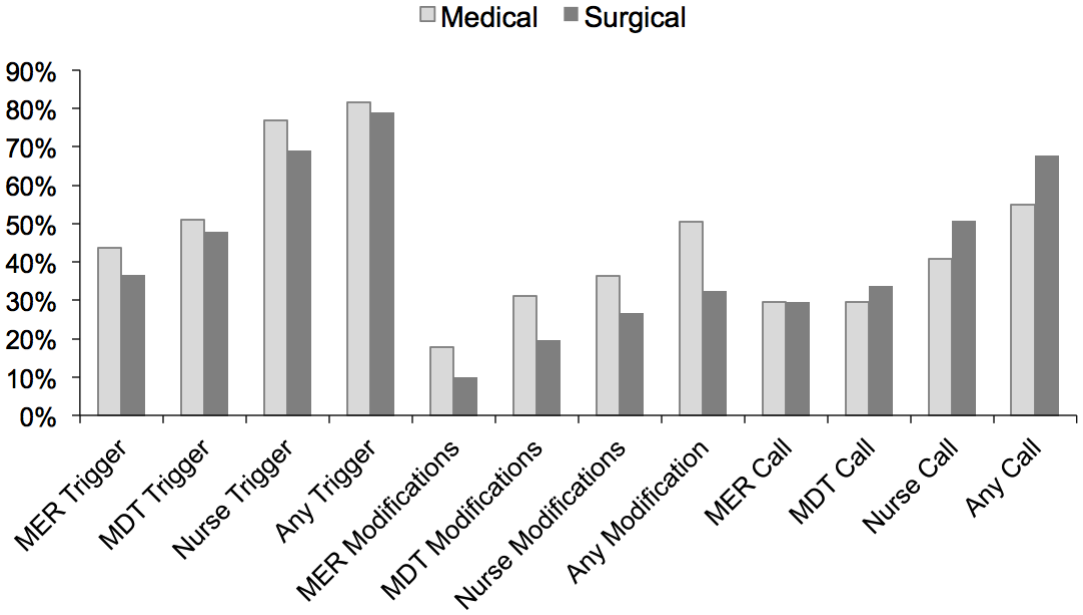
The order of occurrence of Triggers during the 24 hour prevalence periods. MER: Medical Emergency Response Team. MDT: Multidisciplinary Team

Medical (n = 135) and surgical (n = 71) patients were just as likely to have a Nurse (104 [77%] vs 49 [69%], p = 0.21), MDT (69 [51.1%] vs 34 [47.9%], p = 0.66) or MER (59 [43.7%] vs 26 [36.6%], p = 0.33) Trigger (Table 1), as well as any type of Call (74 [54.8%] vs 48 [67.6%], p = 0.08). In contrast, medical patients had more Modifications overall (68 [50.4%] vs 23 [32.4%], p = 0.01), but a similar proportion of MER (24 [17.8%] vs 7 [9.9%], p = 0.13), MDT (42 [31.1%] vs 14 [19.7%], p = 0.08) and Nurse (49 [36.3%], vs 19 [26.8%], p = 0.17) Modifications (Fig 2).

Patients with (n = 69), compared to those without (n = 137) an NFR order were more likely to have a Trigger (61 [88.4%] vs 105 [76.6%], p = 0.04) a Modification (49 [71%] vs 42 [30.7%], p<0.01) but not a Call (46 [66.7%] vs 76 [55.5%], p = 0.12).

Patients who died (N = 29), compared to survivors (N = 177) of their hospital stay, were just as likely to have any type of Trigger (26 [89.7%] vs 140 [79.1%], P = 0.18), in particular, more MER and MDT Triggers (Table 2). They were also more likely to have a Modification (19 [65.5%] vs 72 [40.7%], p<0.01), in particular a MER (14 [48.3%] vs 17 [9.6%], p<0.01), and MDT Modification (14 [48.3%] vs 42 [23.7%], p<0.01) but not Nurse Modification (11 [37.9%] vs 57 [32.2%], P = 0.543). Patients who died, compared to survivors, were just as likely to have a Call (20 [69%] vs 102 [57.6%], P = 0.25).

Amongst these 206 patients, and over their entire hospital stay, there were a total of 3440 Triggers, of which 2330 (67.7%) were a Nurse, 825 (24%) MDT and 285 (8.3%) MER Triggers. Of these, 1058 (30.8%) of all Triggers, 624 (26.8%) Nurse, 314 (38.1%) MDT and 120 (42.1%) MER Triggers were modified so as not to initiate a Call.

There were 140 (68%) patients in whom a Trigger was not modified, but then did not initiate a Call. This was associated with a nurse Trigger for 128 (62.1%), a MDT Trigger for 67 (32.5%) and a MER Trigger for 30 (14.6%) patients. There were 1706 Nurse, 511 MDT and 165 MER Triggers that were not modified, and thus expected to initiate a Call. Of these, a failure to initiate a Call occurred for 1323 (77.5%) Nurse, 331 (64.8%) MDT, 66 (40%) MER Triggers, and 1720 (72.2%) of all un-modified Triggers. Patients who died, compared to those who survived, where just as likely to have an episode of failure to initiate a Call (23 [79.3%] vs 117 [66.1%], P = 0.16), but more likely for that to be a MER Call (8 [27.6%] vs 22 [12.4%], P = 0.03).

Table 3 illustrates the frequency, and proportion, of Modifications, Calls and Triggers for which an escalated response was not initiated. Triggers that were not documented as ever having occurred (chest pain, urine < 30mls in 4 hours, MDT worried, threatened airway, significant bleeding, no MDT attendance within 30 minutes, seizure, and behavioural disturbance), have been omitted. The number of Modifications, Calls and episodes of failure to initiate a Call, correlated strongly with the total number of Nurse (0.915 [p<0.01], 0.516 [p<0.01], 0.998 [p<0.01]), MDT (0.939 [p<0.01], 0.812 [p<0.01], 0.963 [p<0.01]), MER (0.981 [p<0.01], 0.926 [p<0.10], 0.937 [p<0.01]) and all Triggers combined (0.912 [p<0.01], 0.631 [p<0.10], 0.988 [p<0.01]).

**Table 3:** Incidence group related Medical Emergency Response (MER), Multidisciplinary (MDT) and Nurse Triggers and associated Modifications, Calls and failure to Call.

Tier	Trigger	Total	Modification	Call	Modification (%)	Call (%)	Failure to Call (%)
MER	Cardiac Arrest	2	0	2	0%	100%	0%
MER	Pulse <40	2	0	2	0%	100%	0%
MER	Worried	2	0	2	0%	100%	0%
MER	Conscious = 3	8	5	3	62.5%	37.5%	0%
MER	Pulse >140	26	13	13	50%	50%	0%
MER	RR > 30	41	20	12	48.8%	29.3%	42.9%
MER	SBP > 200	41	12	16	29.3%	39%	44.8%
MER	SaO2 <90%	65	31	12	47.7%	18.5%	64.7%
MER	SBP <90	98	39	36	39.8%	36.7%	39%
MER	Total	285	120	99	42.1%	34.7%	40%
MDT	Temp <35	4	1	1	25%	25%	66.7%
MDT	RR = 8–10	7	5	1	71.4%	14.3%	50%
MDT	O2 flow >6L/min	9	4	3	44.4%	33.3%	40%
MDT	Pulse = 40–50	21	13	2	61.9%	9.5%	75%
MDT	Temp > 38.6	30	1	9	3.3%	30%	69%
MDT	Pain Score = 8–10	69	3	36	4.3%	52.2%	45.5%
MDT	RR = 26–30	122	64	26	52.5%	21.3%	55.2%
MDT	Pulse = 120–140	124	65	27	52.4%	21.8%	54.2%
MDT	SBP = 180–200	176	54	43	30.7%	24.4%	64.8%
MDT	SBP = 90–100	263	104	32	39.5%	12.2%	79.9%
MDT	Total	825	314	180	38.1%	21.8%	64.8%
Nurse	Worried	2	0	2	0%	100%	0%
Nurse	Temp = 35.1–35.5	17	0	4	0%	23.5%	76.5%
Nurse	O2 flow = 6L/min	59	13	7	22%	11.9%	84.8%
Nurse	Temp = 38.1–38.6	70	7	16	10%	22.9%	74.6%
Nurse	Conscious = 2	81	7	11	8.6%	13.6%	85.1%
Nurse	SBP = 170–180	150	28	26	18.7%	17.3%	78.7%
Nurse	Pulse = 50–60	176	49	24	27.8%	13.6%	81.1%
Nurse	Pain Score = 5–7	314	0	154	0%	49%	51%
Nurse	Pulse = 100–120	381	117	51	30.7%	13.4%	80.7%
Nurse	RR = 21–25	422	151	38	35.8%	9%	86%
Nurse	SaO2 = 90–94%	658	252	50	38.3%	7.6%	87.7%
Nurse	Total	2330	624	383	26.8%	16.4%	77.5%
All Tiers	Total	3440	1058	662	30.8%	19.2%	72.2%

Modification: proportion of all Triggers that were modified.

Call: proportion of all Triggers that were a Call

Failure to Call: proportion of all Triggers that were not modified, but did not progress to a Call

MER: Medical Emergency Response Team

MDT: Multidisciplinary Team

## Discussion

### Summary of findings

Almost 50% of general medical and surgical ward patients have a Trigger for an escalated response (a Call) throughout a 24 hour period and 80% have a Trigger throughout their entire hospital stay. Not all Triggers generated a Call as intended (Afferent Limb Failure). This was because many were either modified, so as to intentionally not initiate a Call, or a decision/omission was made to not Call. The prevalence and incidence of failing to initiate a Call was higher than that of Modifications. The number of failures to initiate a Call and of Modifications correlated strongly with the number of Triggers.

### Comparison with other studies

The prevalence of Triggers at the highest level of escalation (MER) was similar to that of other studies [18,19]. In addition there was a high prevalence/incidence of Triggers at the lower tiers of escalation. The consequences of this would have been an additional workload upon ward-based nursing and medical staff. As a comparison, median scene times for RRT calls have been recorded to be 17–20 minutes [20]. This additional burden, upon an already high medical and nursing workload, has the potential for adverse consequences for both patients and staff [21–24]. In particular as the predictive value of vital signs at lower levels of patient illness acuity is low [25].

The prevalence and incidence of Modifications to pre-defined Triggers has not been documented before. They were disturbingly high, potentially eroding the structured design and the escalation response features of this observation and response chart. Modifications were common across all tiers, including MER Triggers, and were equally prevalent amongst medical and surgical patients, and patients with an NFR. The incidence of Modifications was higher amongst medical patients, and the incidence of Calls higher for surgical patients, despite a similar incidence of Triggers for both medical and surgical patients. These variations may be explained by the findings that medical patients were older and more likely to have NFR orders, and thus, more likely to have abnormal vital signs [18], fewer critical care interventions by RRT [20], or modifications made to their RRT Triggers [26]. The incidence of Modifications was higher amongst patients who died compared to survivors. However we cannot draw a direct link to mortality as over 90% of patients who died had an NFR order. Further investigation is required to better explain these variations.

Modifications, and failure to initiate a Call, varied according to the type of Trigger. As expected, a lower physiological trigger threshold would be more sensitive at triggering a Call, but be less specific for an adverse event [25]. Modifications and failure to initiate a Call were common, particularly within the lower tiers, and the more frequently occurring Triggers. This may reflect an element of “alarm fatigue” [27–29]. Alarm fatigue with respect to electronic patient monitors, is common, and hazardous to patients, as well as disengages staff with the monitor, who may also ignore and/or modify alarm settings [29–30]. The adverse consequences of a failure to call, ie Afferent Limb Failure (ALF) in respect to RRT (equivalent to our MER team) are well recognised [3,7]. Little is known however about the consequences of ALF for a lower tier response.

The type of escalation response to deteriorating patients varies amongst health jurisdictions. Multi-tiered responses are based upon the assumption that a “lower-level” clinical response, set at a lower trigger threshold, may prevent further patient deterioration and thus avoid the need for a “higher-level” response or adverse event. There is little evidence that a graded, multi-tiered response, based upon escalating trigger thresholds, in contrast to single-tiered response, is associated with improved patient outcomes. Our observation of the sequence of Trigger occurrence did not reveal a predominate pattern of “escalation”. For example, a Nurse:MDT:MER, or Nurse:MDT Trigger sequence was no more common than a MDT:Nurse or MER:MDT:Nurse Trigger sequence. It may be argued that we observed fewer MER and MDT triggers because of the larger number of Nurse Triggers. However 35% of Nurse Triggers were modified to not generate an escalated response, and 75% of Triggers not modified, did not result in an escalated response.

### Strengths and Weaknesses

The study population was restricted to general medical and surgical patients. Our findings may not be generalizable to other patient populations, or health jurisdictions. However studies have identified similarities in patient demographics and triggers for a RRT call across different health jurisdictions [31]. Our incidence sample was not randomly selected and this may have introduced a bias, however they were of similar age and gender distribution to the prevalence group. Our study was undertaken three months following chart implementation. It is possible that staff chart unfamiliarity may have contributed to our findings.

### Implications for clinicians and policy makers

Our study is the first to explore prevalence and incidence of Triggers, Modifications, Calls and failure to Call, within a multi-tiered observation and response chart. It identifies important findings in respect to potential risks associated with the additional imposed workload, and the risk of failing to comply with, what is a very basic and important aspect of routine patient care, being, the recording and responding to patient observations. The occurrence, and consequences of, Modifications, and a failure to Call, in response to Triggers requires on going evaluation. It also sets a foundation for further studies in investigating the utility, and consequences of, Modifications and ALF for tiers of response other than for a RRT.

In summary, we found that, in association with a multi-tiered observation and response chart, Triggers, Modifications of Triggers, and failure to Call, are common. The overall number of Triggers may disrupt clinical workload. Modifications may erode the structured design, and escalation response features for the detection, and response to, the acutely deteriorating patient, of an observation and response chart. Modifications, and failure to Call, have the potential to cause patient harm and correlate strongly to the number of Triggers. Further investigation is required to ascertain the clinical efficacy and actual patient benefits, of observation and response charts, in contrast to their potential burden upon clinical workload, propensity for modifications and the risks of associated failure to initiate a Call, particularly amongst the lower tiers of response.

## References

[1] Buist M, Bernard S, Nguyen TV, Moore G, Anderson J. Association between clinical abnormal observations and subsequent in-hospital mortality: a prospective study. Resuscitation. 2004;62:137–41. 15294398 10.1016/j.resuscitation.2004.03.005

[2] Hillman K, Bristow PJ, Chey T, Daffurn K, Jacques T, Norman SL et al Antecedents to hospital deaths. Intern Med J 2001;31:343–8. 11529588 10.1046/j.1445-5994.2001.00077.x

[3] Trinkle RM, Flabouris A. Documenting Rapid Response System Afferent Limb Failure and Associated Patient Outcomes. Resuscitation 2011;82:810–14. 10.1016/j.resuscitation.2011.03.019 21497982

[4] Trinkle R, Flabouris A. Critical Care Patient Reviews Preceding Adverse Events: Their Nature and Impact upon Patient Outcome. Resuscitation 2011;82:810–814 21497982

[5] Winters BD, Weaver SJ, Pfoh ER, Yang T, Pham JC, Dy SM. Rapid-response systems as a patient safety strategy: A systematic review. Ann Intern Med 2013;158:417–25. 10.7326/0003-4819-158-5-201303051-00009 23460099 PMC4695999

[6] Australian Commission on Safety and Quality in Health Care (ACSQHC) (9 2011), National Safety and Quality Health Service Standards, ACSQHC, Sydney

[7] Boniatti M. M, Azzolini N, Viana M. V, Ribeiro BS, Coelho RS, Castilho RK et al Delayed medical emergency team calls and associated outcomes. Crit Care Med, 2014;42:26–30. 10.1097/CCM.0b013e31829e53b9 23989173

[8] Casamento AJ, Dunlop C, Jones DA, Duke G. Improving the documentation of medical emergency team reviews. Crit Care Resusc 2008;10:29–34. 18304014

[9] Cretikos MA, Chen J, Hillman KM, Bellomo R, Finfer SR, Flabouris A et al The effectiveness of implementation of the medical emergency team (MET) system and factors associated with use during the MERIT study. Crit Care Resusc 2007;9:206–12 17536993

[10] Van Leuvan C, Mitchell I. Missed opportunities? An observational study of vital sign measurements. Crit Care Resusc 2008;10:111–5. 18522524

[11] Azzopardi P, Kinney S, Moulden A, Tibballs J. Attitudes and barriers to a Medical Emergency Team system at a tertiary paediatric hospital. Resuscitation. 2011;82:167–74 10.1016/j.resuscitation.2010.10.013 21106289

[12] Chen J, Bellomo R, Flabouris A, Hillman K, Finfer S, the MERIT Study Investigators for the Simpson Centre et al The relationship between early emergency team calls and serious adverse events. Crit Care Med 2009;33:148–53 10.1097/CCM.0b013e3181928ce3 19050625

[13] Chatterjee MT, Moon JC, Murphy R, McCrea D. The "OBS" chart: an evidence based approach to re-design of the patient observation chart in a district general hospital setting. Postgrad Med J 2005;81:663–6. 16210466 10.1136/pgmj.2004.031872 PMC1743374

[14] Preece MHW, Hill A, Horswill MS, Watson MO. Supporting the detection of patient deterioration: Observation chart design affects the recognition of abnormal vital signs. Resuscitation 2012;83:1111–1118. 10.1016/j.resuscitation.2012.02.009 22353643

[15] Jenkins PF, Thompson CH, MacDonald AB. What does the future hold for general medicine? Med J Aust. 2011;195:49–50. 21728946 10.5694/j.1326-5377.2011.tb03192.x

[16] Prytherch D, Senapati A, O'Leary D, Thompson MR. Sub-specialization in general surgery: the problem of providing a safe emergency general surgical service. Colorectal Dis. 2006;8:273–7 16630229 10.1111/j.1463-1318.2005.00932.x

[17] Department of Health and Ageing. Government of South Australia. Recognising and Responding to Clinical Deterioration Brochure 2013. http://www.sahealth.sa.gov.au/wps/wcm/connect/e0a9a500424c28499decfdef0dac2aff/Rec%26ResClinicalDeteriorationBrochure_PHCS_SQ_20131216.pdf?MOD=AJPERES&CACHEID=e0a9a500424c28499decfdef0dac2aff. Accessed Dec 2014

[18] Bucknall TK, Jones D, Bellomo R, Staples M, the RESCUE Investigators. Responding to medical emergencies: System characteristics under examination (RESCUE). A prospective multi-site point prevalence study. Resususcitation 2013;84:179–183. 10.1016/j.resuscitation.2012.06.015 22771869

[19] Bell MA, Konrad D, Granath F, Ekbom A, Martliong CR. Prevalence and sensitivity of MET-criteria in a Scandinavian University Hospital. Resuscitation 2006;70:66–73. 16757089 10.1016/j.resuscitation.2005.11.011

[20] Coventry C, Flabouris A, Sundararajan K, Cramey T. Rapid Response Team calls to patients with a pre-existing Not for Resuscitation order. Resuscitation 2013;84:1035–9 10.1016/j.resuscitation.2013.01.021 23376582

[21] Patrician PA, Loan L, McCarthy M, Fridman M, Donaldson N, Bingham M et al The association of shift-level nurse staffing with adverse patient events. J Nurs Adm 2011;41:64–70. 10.1097/NNA.0b013e31820594bf 21266884

[22] Ball JE, Murrells T, Rafferty AM, Morrow E, Griffiths P. 'Care left undone' during nursing shifts: associations with workload and perceived quality of care. BMJ Qual Saf 2014;23:116–25. 10.1136/bmjqs-2012-001767 23898215 PMC3913111

[23] Ausserhofer D, Zander B, Busse R, Schubert M, De Geest S, Rafferty AM et al Prevalence, patterns and predictors of nursing care left undone in European hospitals: results from the multicountry cross-sectional RN4CAST study. BMJ Qual Saf 2014;23:126–35. 10.1136/bmjqs-2013-002318 24214796

[24] Elliott DJ, Young RS, Brice J, Aguiar R, Kolm P. Effect of hospitalist workload on the quality and efficiency of care. JAMA Intern Med 2014;174:786–93. 10.1001/jamainternmed.2014.300 24686924

[25] Cretikos M, Chen J, Hillman K, Bellomo R, Finfer S, Flabouris A et al The Objective Medical Emergency Team Activation Criteria: A Case-Control Study. Resuscitation 2007;73:62–72. 17241732 10.1016/j.resuscitation.2006.08.020

[26] Sundararajan K, Flabouris A, Keeshan A, Cramey T. Documentation of limitation of medical therapy at the time of a Rapid Response Team call. Aust Health Rev. 2014 5;38:218–22. 10.1071/AH13138 24589293

[27] Varpio L, Kuziemsky C, MacDonald C, King WJ. The helpful or hindering effects of in-hospital patient monitor alarms on nurses: a qualitative analysis. Comput Inform Nurs 2012;30:210–7. 10.1097/NCN.0b013e31823eb581 22156767

[28] Graham KC, Cvach M. Monitor alarm fatigue: standardizing use of physiological monitors and decreasing nuisance alarms. Am J Crit Care. 2010;19:28–34. 10.4037/ajcc2010651 20045845

[29] Cvach M. Monitor alarm fatigue: an integrative review. Biomed Instrum Technol. 2012;46:268–77. 10.2345/0899-8205-46.4.268 22839984

[30] Schmid F, Goepfert M S, Kuhnt D, Eichhorn V, Diedrichs S, Reichenspurner H et al The Wolf Is Crying in the Operating Room: Patient Monitor and Anesthesia Workstation Alarming Patterns During Cardiac Surgery. Anesth Analg 2011;112:78–83. 10.1213/ANE.0b013e3181fcc504 20966440

[31] Jäderling G, Calzavacca P, Bell M, Martling CR, Jones D, Bellomo R et al The deteriorating ward patient: a Swedish-Australian comparison. Intensive Care Med 2011;37:1000–1005 10.1007/s00134-011-2156-x 21369815

